# Quantitatively Detecting Camellia Oil Products Adulterated by Rice Bran Oil and Corn Oil Using Raman Spectroscopy: A Comparative Study Between Models Utilizing Machine Learning Algorithms and Chemometric Algorithms

**DOI:** 10.3390/foods13244182

**Published:** 2024-12-23

**Authors:** Henan Liu, Sijia Ma, Ni Liang, Xin Wang

**Affiliations:** School of Physical Science and Technology, Tiangong University, Tianjin 300387, China

**Keywords:** adulteration quantification, camellia oil products, Raman spectroscopy, back propagation neural network, rice bran oil products, corn oil products

## Abstract

The fast and accurate quantitative detection of camellia oil products is significant for multiple reasons. In this study, rice bran oil and corn oil, whose Raman spectra both hold great similarities with camellia oil, are blended with camellia oil, and the concentration of each composition is predicted by models with varying feature extraction methods and regression algorithms. Back propagation neural network (BPNN), which has been rarely investigated in previous work, is used to construct regression models, the performances of which are compared with models using random forest (RF) and partial least squares regression (PLSR). Independent component analysis (ICA), competitive adaptive reweighing sampling (CARS), and their dual combinations served to extract spectral features. In camellia oil adulteration with rice bran oil, both the ICA-BPNN and ICA-PLSR models are found to achieve satisfactory performances. For camellia oil adulteration with rice bran oil and corn oil, on the other hand, the performances of BPNN-based models are substantially deteriorated, and the best prediction accuracy is achieved by a PLSR model coupled with CARS-ICA. In addition to performance fluctuations with varying regression algorithms, the output for feature extraction method also played a vital role in ultimate prediction performance.

## 1. Introduction

Camellia oil is a type of edible oil extracted from camellia seeds. Being remarkably high in oleic acids and unsaturated fatty acids, camellia oil has a very similar fatty acid composition to olive oil, thus being dubbed as “oriental olive oil” [1,2]. The regular consumption of a camellia oil-rich diet is capable of not only lowering cholesterol levels, but is also able to reduce the probability of cardiovascular diseases [3]. Moreover, as a rich source of antioxidants, camellia oil can effectively prevent liver damage and gastrointestinal ulcers [4], and provide inhibitory effects on cancer-inducing virus [5]. The high nutritional value of camellia oil makes the oil a very good target for adulteration, which not only endangers the economic interests of the consumers, but also poses potential health risks to them. It is, therefore, increasingly urgent to develop a reliable and rapid method to quantitatively detect camellia oil-targeted adulteration.

Various analytical methods have been used to analyze camellia oil adulteration. Chromatography and mass spectrometry, which are probably the mostly used techniques in the field, have been utilized individually or in combined manners to provide predictions with high specificity and sensitivity [6]. However, for these techniques, the sample preparation is generally extensive, and the analysis process is always tedious and invasive. The spectroscopic techniques (involving Raman, IR, etc.), on the other hand, are receiving increasing attention since these techniques usually require only minimal sample pre-treatments and the measurements are easy to handle. Remarkably, these techniques are also non-invasive, which is very appealing for food-related analysis. Among the spectroscopic techniques, Raman spectroscopy is one of the most promising techniques for oil adulteration detection. In addition to advantages such as being fast and non-invasive, Raman spectroscopy demonstrates a very high sensitivity when discriminating materials of similar structures or compositions, and holds great potential to realize instrumental miniaturization. In addition, the technique has been reported to be highly sensitive to lipids [7], which makes the technique especially appropriate to analyze edible oil adulteration [8,9,10,11].

It is necessary to combine Raman spectroscopy with certain algorithms to reveal the relationship between spectra and the corresponding chemical compositions. In addition to traditional chemometric algorithms such as partial least squares regression (PLSR) and linear discriminant analysis (LDA) [12,13], algorithms originating from machine learning are currently receiving increasing attention due to their superiorities in data analysis. Taking back propagation neural network (BPNN) as an instance, it is widely used to solve complex nonlinear problems with strong approximation and generalization abilities. Being excellent in dealing with data of large volume and high dimension, BPNN is a very promising algorithm to realize spectra-based quantitative analysis [14].

Currently, however, there are only a very limited number of quantitative investigations on camellia oil adulteration using algorithms such as BPNN [15,16]. Furthermore, in the ongoing research on camellia oil-targeted adulteration, only limited oil species are considered [6]. In the previous work, adulterations by oil species including sunflower oil, soybean oil, and corn oil have been investigated [17,18,19,20]. However, adulteration with rice bran oil, of which the Raman spectra is very similar to that of camellia oil and the price is substantially lower, is scarcely considered [6].

In this work, with the help of Raman spectroscopy, a study to quantitatively detect binary and ternary camellia oil adulteration is conducted. Different algorithms including BPNN, random forest (RF), and PLSR are individually utilized to establish regression models in combination with feature extraction methods, including independent component analysis (ICA), competitive adaptive reweighing sampling (CARS), and their dual combinations. By doing this, we are not only able to find the optimal model to predict the adulteration rates, but we can also explore and compare the performance differences when the algorithm varies. A commercial Raman spectrometer is used to collect the spectra, which are then properly pre-processed before further analyses. Based on the principal component analysis (PCA) in this work, rice bran oil and corn oil demonstrate the largest similarities with camellia oil among all the oil species that have been characterized. For the sake of brevity, camellia and rice bran blended oil (abbreviated as camellia–rice oil in the following contents) is selected to be the studied binary adulterated oil, since when compared to it, there exists a considerable amount of work already conducted on camellia–corn blended oil products [12,21]. The blended oil obtained by mixing corn oil and rice bran oil into camellia oil (abbreviated as camellia–corn–rice oil in the following contents), on the other hand, serves as the ternary blended oil to be analyzed. The prediction results for binary and ternary adulteration are analyzed and compared accordingly, and the optimal regression models are found for each case.

## 2. Materials and Methods

### 2.1. Sample Preparation

Pure edible oil products including camellia oil, rice bran oil, corn oil, walnut oil, sunflower oil, sesame oil, and soybean oil were purchased at local supermarkets. Three pure oil samples were prepared for each oil species. Binary blended-oil samples were prepared by mixing camellia oil and rice bran oil accordingly. A total of 37 different adulteration rates were prepared, with a gradient of 2% when the concentration of rice bran oil (the adulterating oil) ranged from 2% to 60%, and a gradient of 5% when the concentration of rice bran oil ranged from 65% to 95%. For each adulteration rate, three samples were prepared, thus resulting in 111 samples in total. Ternary blended-oil samples, on the other hand, were prepared by mixing camellia oil, corn oil, and rice bran oil accordingly. The total adulteration concentration ranges from 10% to 50%, with a gradient of 5%. When the total adulteration was fixed, the individual concentration of rice bran oil and corn oil were altered, resulting in a set of 4 different concentration rates. For each adulteration rate, 2 samples were prepared, thus resulting in 72 samples in total. When preparing the blended-oil samples, each compositional oil product was pipetted and transferred to beakers which had been washed thoroughly with deionized water and dried afterwards. Subsequently, the beakers were placed on a magnetic stirrer (Surui Instruments, Changzhou, China) and vortexed for 5 min to ensure even oil mixing. All of the samples were stored in a cold and dark environment before spectral collection. When Raman measurements were to be conducted, 0.1 mL oil for each sample was extracted using a pipette and dropped onto a glass slide.

### 2.2. Raman Spectra Collection

In this work, a commercial Raman spectrometer (Horiba Scientific, Kyoto, Japan) was used for spectral collection. The excitation wavelength was 532 nm, with a maximum power of 100 mW which could be delicately tuned by using a set of built-in neutral density filters. A long-working distance objective lens was used to collect the Raman signals and its magnification was 50×. The spectral range was 300–3200 cm^−1^ and the spectral resolution was 0.5 cm^−1^. For each collected spectrum, the integration time was 20 s and the accumulation number was 2 to ensure a good signal-to-noise ratio (SNR). The inevitable non-uniformness in focus position was considered, and the spectra of each sample were collected at 10 randomly selected positions and averaged afterwards. Thus, we have a total of 1110 and 720 spectra for the binary and ternary adulteration case, respectively. When subjected to modeling, the whole set of spectra was divided into a training set and a testing set in a 3:1 ratio, and the validation set was extracted from the training set by 10-fold cross-validation [22,23].

### 2.3. Raman Data Pre-Processing

The collected spectra are often affected by interference factors originating from the light source, the instrumental noise, and the fluorescent backgrounds, resulting in noise and baseline drift in the raw data. Thus, it is necessary to perform pre-processing before further analysis. The multiplicative scatter correction (MSC) [24,25] was used to remove the interference from scattering, and a polynomial fitting method was used to remove the fluorescence backgrounds. Subsequently, the spectra were further processed by the Savitzky–Golay (S-G) [26,27] convolution smoothing to further reduce the interference from the noise. The corresponding formulas are listed as follows:

(1) $$X_{MSC}=\frac{X-\bar{X}}{\sqrt{\frac{\sum_{k=1}^{m}{\left(X_{k}-\bar{X}\right)}^{2}}{m-1}}}$$

where m is the number of variables and $\bar{X}$ is the average for all spectra.

(2) $$x_{k,\;\;S-G}={\bar{x}}_{k}=\frac{1}{H}\sum_{i=-\omega }^{+\omega }x_{k+i}h_{i}$$

where $h_{i}$ is the smoothing coefficient and H is the normalization factor, $H=\sum_{i=-\omega }^{+\omega }h_{i}$.

### 2.4. Spectral Feature Extraction

Multi-collinearity and redundant information in original spectra substantially lower modeling efficiency and deteriorate model accuracy and robustness. Feature extraction will effectively solve these issues, which makes itself a necessity in quantitative modeling. The algorithms used in this work include PCA, ICA, and CARS. While PCA was solely used to visualize the vibrational distinctions among different oil species, the other two algorithms were used to aid quantitative modeling. In the ternary case, ICA and CARS were combined to realize dual feature extraction to further improve the regression performances. The software used in this work was Python 3.9 for spectra pre-processing, feature extraction and modeling.

PCA projects the original data into a new coordinate system of lower dimension by linear transformation and seeks covariance maximization. By obtaining the eigenvalues and vectors of the covariance matrix, vectors corresponding to the top eigenvalues are selected since they are most representative of the original data [28,29,30].

ICA is aimed to extract mutually statistically independent components by linear transformation. Opposite to methods such as PCA, ICA seeks the maximization in statistical independence for the resulting components, which generally offers a better demonstration of data structure and hidden features [31,32].

CARS is a method utilizing the “survival of the fittest” principle. By adaptively reweighted sampling, wavelength variables with large absolute regression coefficients are selected, while variables with trivial coefficients are removed. The optimal subset of variables is determined by cross-validation, since it is expected to minimize the root mean square error (RMSE) [33,34].

### 2.5. Modeling Algorithms

#### 2.5.1. Back Propagation Neural Network (BPNN)

**Figure 1:**
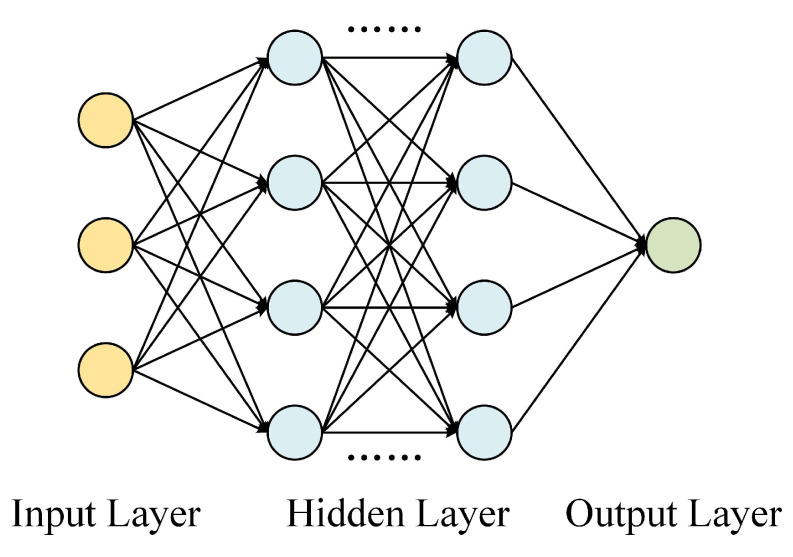
The structure of back propagation neural network (BPNN).

BPNN is a widely used feedforward neural network which consists of an input layer, a series of hidden layers, and an output layer. As the algorithm’s most distinctive feature, the signals propagate forwards in the network while the errors propagate backwards [16]. By continuously adjusting the weights and the neuron bias, the actual outputs gradually approach the expected ones after a number of iterations [35]. The structure of BPNN is shown in Figure 1.

Despite its advantages, however, BPNN also suffers from severe disadvantages such as being easily to fall into local optimum. In this work, the gradient descent-based Levenberg–Marquardt (LM) algorithm is used to optimize the BPNN method [36]. Combined with the Newton method, the modeling strategy is adaptively adjusted, which not only circumvents the fatal shortcoming of BPNN, but also realizes a faster convergence speed [37].

The weight and the threshold adjustment formula for LM-coupled BPNN are listed as follows [38]:

(3) $$\Delta W=-{\left[J^{T}\left(X\right)\cdot J\left(X\right)+U\cdot I\right]}^{-1}J^{T}\left(X\right)\cdot e\left(X\right)$$

(4) $$W_{k+1}=W_{k}-{\left[J^{T}\left(X\right)\cdot J\left(X\right)+U\cdot I\right]}^{-1}J^{T}\left(X\right)\cdot e\left(X\right)$$

(5) $$e\left(x\right)={\left[e_{1}\left(x\right)\cdot e_{2}\left(x\right)...e_{i}\left(x\right)\right]}^{T}$$

where X is the input of BPNN, $W_{K}$ represents the weight vector of the *k*th iteration, I is the identity matrix, J(X) is the Jacobian matrix, U is a non-negative value, and $e_{i}\left(x\right)$ indicates the error between the network output and the actual value.

#### 2.5.2. Partial Least Squares Regression (PLSR)

Roughly speaking, PLSR is a “modified” version of least squares regression in the latent space, used to tackle with the singularity of covariance matrix [39,40,41]. The latent variables, or PLSR factors, are extracted from the original data, which are then used in multiple linear regression to find out the relationship between the original spectral variables and the responses. PLSR is especially advantageous in cases where the number of variables is considerably larger than the number of samples, or where multi-collinearity exists in the variables.

#### 2.5.3. Random Forest (RF)

RF, being an ensemble of decision trees, is a type of un-biased machine learning algorithm [40,42]. In the case of regression, the predicted result is the average of each tree in the forest. In the training process, decision trees are constructed by randomly selected subsets of data, and the corresponding performance is evaluated by cross-validation. Generally, the number of trees, the number of predictors to select at each node, and the minimum size of the nodes need to be tuned. In this work, the number of decision trees is determined to be 33 by multi-parameter debugging.

### 2.6. Model Evaluation Metrics

For regression models, the coefficient of determination (*R*^2^) and RMSE are commonly used evaluation metrics. *R*^2^ measures the correlation between the predicted value and the actual value, and a *R*^2^ close to 1 indicates the model’s good prediction of the target data. The calculation formula is listed as follows:

(6) $$R^{2}=1-\frac{\sum_{i=1}^{n}{\left(y_{i}-{\hat{y}}_{i}\right)}^{2}}{\sum_{i=1}^{n}{\left(y_{i}-\bar{y}\right)}^{2}}$$

where y represents the true value of the sample, $\hat{y}$ represents the predicted value, $\bar{y}$ represents the average of the true values, and n is the number of samples.

RMSE measures the prediction error of the model, and the closer its value is to 0, the better. The calculation formula is listed as follows:

(7) $$RMSE=\sqrt{\frac{1}{n}\sum_{i=1}^{n}{\left(y_{i}-{\hat{y}}_{i}\right)}^{2}}$$

## 3. Results and Discussion

### 3.1. Raman Spectra

**Figure 2:**
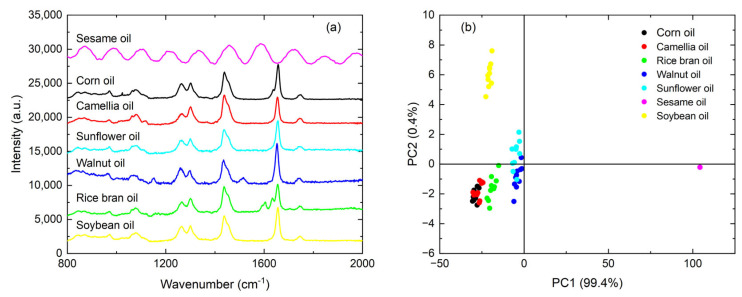
A comparison of seven edible oil species: (**a**) pre-processed Raman spectra, and (**b**) scatterplot of projections for the first two principal components on a 2D plane.

Figure 2a presents the pre-processed Raman spectra for seven species of pure edible oil. Some distinctive features are demonstrated depending on the oil species: for instance, walnut oil shows a quite evident peak at 1150 cm^−1^, which is absent in the other oil species. These distinctive features, however, are much weaker as compared to the spectral commonness. Except for sesame oil, all the other oil species show very similar spectral features in the 1200–1340 cm^−1^ range, the 1400–1500 cm^−1^ range, and the vicinity of the 1654 cm^−1^ peak, which are all strong spectral features. However, there are also nontrivial relative differences in the peak intensities, for instance, the 1265 cm^−1^ peak, the 1300 cm^−1^ peak, and the vicinity of the 1654 cm^−1^ peak.

PCA is conducted on the oil species in Figure 2a for quick discrimination, and Table S1 lists the percentage of variance for each principal components and their cumulative variances. When extracting the first two principal components, the cumulative variance contribution rate exceeds 90%, indicating that they carry the vast majority of the information in the Raman spectra. Based on the PCA results shown in Figure 2b, most of the oil species can be clearly distinguished, except for camellia oil, rice bran oil, and corn oil, whose PCA projections are located quite closely to each other, demonstrating the substantial similarity in their Raman spectra. Thus, it will be quite challenging to quantitatively analyze camellia–rice and camellia–corn–rice blended-oil samples.

**Figure 3:**
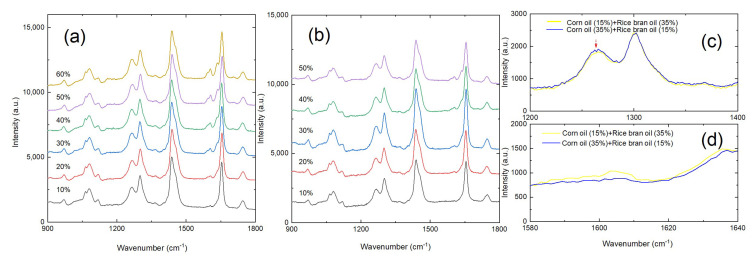
Representative Raman spectra for (**a**) camellia–rice blended-oil samples and (**b**) camellia–corn–rice blended-oil samples with varying adulteration rates. Note that in panel (**b**), it is the total adulteration rate that is noted in the plot. (**c**,**d**) are zoom-ins of the different spectral ranges for two spectra of the camellia–corn–rice oil sample when the total adulteration rate reaches 50%. The adulteration rate for rice bran oil and corn oil are noted accordingly.

Representative Raman spectra for the camellia–rice and camellia–corn–rice blended-oil samples with varying adulteration rates are shown in Figure 3, while the whole set of the spectra can be referred to in the Supplementary Materials (SM) as Figures S1 and S2. For the binary blended-oil samples in panel (a), no matter what the concentration rate is, they always demonstrate very similar spectral profiles. However, as the concentration of rice bran oil increases, the intensities of the 1600 cm^−1^ peak and the 1635 cm^−1^ peak apparently increase. For the ternary blended-oil samples in panel (b), spectral variations could also be tracked in certain ranges. Panels (c) and (d) show the comparison of two Raman spectra, for which they share the same total adulteration rate (50%) but differ in the individual adulteration rates for rice bran oil and corn oil. When the concentration of corn oil increases, an enhancement at the 1265 cm^−1^ peak and an attenuation at the 1605 cm^−1^ peak can be simultaneously observed. Oppositely, the increasing concentration of rice bran oil is readily revealed by the enhancement at the 1605 cm^−1^ and the 1635 cm^−1^ peak. The above observations ensure that Raman spectroscopy could be effectively used to quantitatively detect camellia–rice and camellia–corn–rice adulterations.

### 3.2. Regression Models

**Figure 4:**
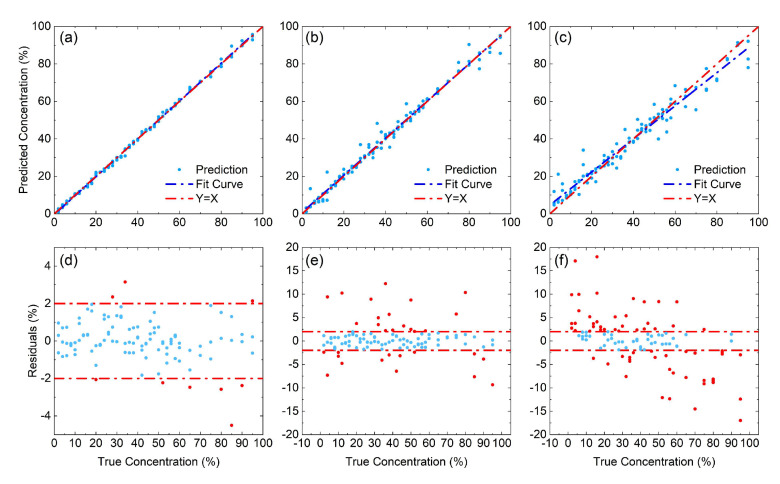
Prediction of adulteration concentration for camellia–rice blended-oil samples using ICA coupled regression models: regression results for (**a**) BPNN, (**b**) PLSR, and (**c**) RF; residual distribution for (**d**) BPNN, (**e**) PLSR, and (**f**) RF. In panels (**d**–**f**), the dash-dotted horizontal lines are for eye guidance purposes. The red (blue) dots show residuals with magnitudes larger (no larger) than 2%.

The regressions of camellia–rice blended-oil samples have been conducted by coupling two feature extraction methods (ICA and CARS) individually with BPNN, PLSR, and RF, respectively. Figure 4 presents the prediction results when ICA is coupled with the three modeling algorithms, and the results by CARS can be referred to by Figure S3 in SM. Taking the ICA-BPNN model as an instance, the predicted concentrations are located in the vicinity of the true ones without any obvious deviation, demonstrating a general linear relationship with a slope of 1. The majority of the errors between true and predicted concentrations, as indicated by panel (d), are within ±2%. Only very limited number of outliers are found, with values within ±5%. When switching to PLSR, the predicted results also follow a linear relationship with a slope close to 1. The residual distributions, however, are found to be more diverse. The errors now present more outliers with larger magnitudes, indicating a little less stability for the PLSR model. When RF is selected, the prediction results further deteriorate. While the deviation between the “Y = X” line (red) and the fitted line for the prediction (blue) is very trivial in panel (a) and (b), the deviation now is non-negligible in panel (c). Such an enlarged deviation is also demonstrated by the increasing number of outliers shown in panel (f).

**Figure 5:**
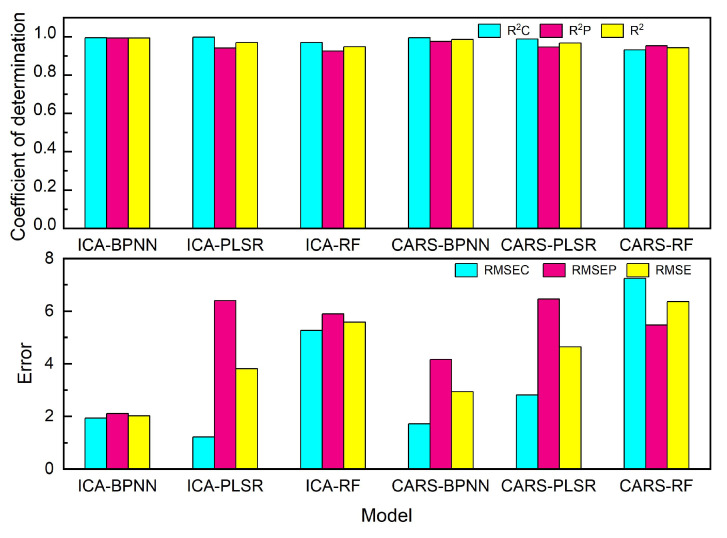
Comparison among different models for camellia–rice blended-oil samples.

The evaluation metrics for the different models are shown in Figure 5, with the values of R^2^ and RMSE listed in Table S2. From the perspective of R^2^, all the BPNN- and PLSR-based models achieve generally satisfactory results, while the performance of RF-based models is slightly inferior. In contrast, the difference from the perspective of prediction errors is larger. The RMSE is generally the smallest in the BPNN-based models, which is somewhat enlarged in the PLSR-based models and further enlarged in the RF-based models. When applying CARS instead of ICA, the corresponding prediction performances are slightly deteriorated. For instance, the R^2^ for ICA-PLSR model is 0.969, which goes to 0.967 when CARS is applied instead, and the RMSE is increased from 3.80 to 4.64. While performance differences are demonstrated by all the models, the BPNN-based models undergo the smallest performance fluctuation when switching from ICA to CARS. Generally speaking, both the ICA-BPNN and the ICA-PLSR model could provide a satisfactory prediction for the binary adulteration issue.

**Figure 6:**
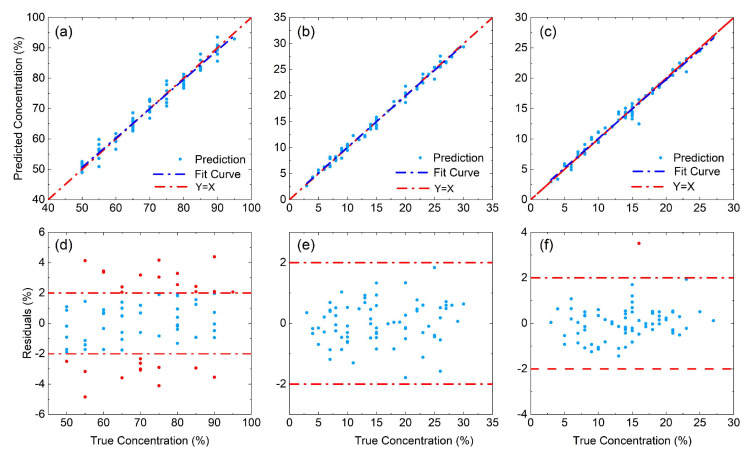
Prediction for camellia–corn–rice blended-oil samples using CARS-ICA-PLSR model: regression results for (**a**) camellia oil, (**b**) corn oil, and (**c**) rice bran oil; residual distributions for (**d**) camellia oil, (**e**) corn oil, and (**f**) rice bran oil. In panels (**d**–**f**), the dash-dotted horizontal lines are for eye guidance purposes. The red (blue) dots show residuals with magnitudes larger (no larger) than 2%.

The quantitative analysis on camellia–corn–rice blended-oil samples is conducted using the same set of regression algorithms, that is, BPNN, PLSR and RF. Slightly different from the binary case, in addition to CARS and ICA, dual feature extraction methods including ICA-CARS and CARS-ICA are also applied to extract characteristic spectral ranges. Figure 6 presents the regression results for each compositional oil (that is, camellia oil, corn oil, and rice bran oil) by PLSR using CARS-ICA. Inspecting the panels (a–c), the predictions for all the three compositional oils are highly accurate, since the predicted results follow a linear relationship with a slope very close to 1. For rice bran oil and corn oil concentrations, the predictions turn out to be slightly more accurate than camellia oil, which is shown by panels (d–f). The errors of the former two compositions are generally within ±2% range, with only one outlier (less than 4%) shown when predicting rice bran oil concentration. On the other hand, the errors to predict camellia oil concentration are a little larger. While the majority of the errors are within the ±2% range, there are now more outliers, but the error magnitude is still no larger than 5%.

Tables S3–S5 list the evaluation metrics for predicting the concentration of camellia oil, corn oil, and rice bran oil using the four extraction feature methods coupled with the three regression algorithms. It is found that in contrast to the binary case, the feature extraction method now plays a vital role in prediction performances. Taking the prediction of corn oil concentration using PLSR as an instance, when ICA is used, the R^2^ is only 0.7549, which is improved to 0.848 when switching to CARS. When the dual feature extraction method CARS-ICA is used, R^2^ is further improved to be 0.989. The above observation turns out to hold in the vast majority of the predictions. Furthermore, the CARS-ICA coupled regression models demonstrate the best accuracy when the oil species varies. Taking BPNN-based models as an instance, the CARS-BPNN model achieves a R^2^ of 0.919 for camellia oil, but the prediction accuracy for corn oil and rice bran oil drastically deteriorates, ending up with a R^2^ of 0.688 and 0.457, respectively. The CARS-ICA counterpart, on the other hand, provides an R^2^ over 0.94 for all the compositional oil species. Through a comparison of the CARS-ICA coupled models, the CARS-ICA-PLSR model is found to provide the best prediction performance. Taking camellia oil as an instance, the R^2^ obtained by PLSR, BPNN, and RF is 0.968, 0.945, and 0.967, respectively. The prediction results for the BPNN and RF algorithm when coupled with CARS-ICA are presented in Figures S4 and S5 in SM.

**Figure 7:**
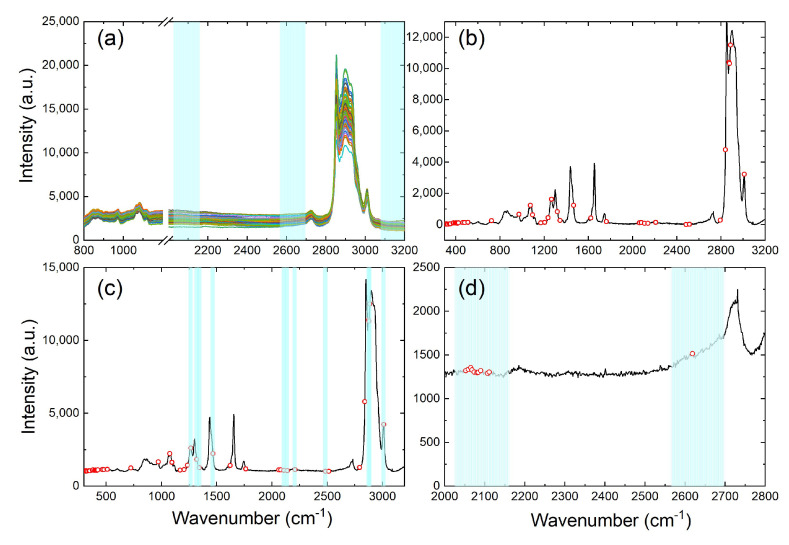
Feature extraction results for camellia–corn–rice blended-oil samples using different methods: (**a**) ICA, (**b**) CARS, (**c**) CARS-ICA, and (**d**) ICA-CARS. Note that the ICA (CARS) selected spectral ranges are indicated by the shadowed areas (red circles). The overlap of the shadowed areas and red circles are the selected spectral variables by the corresponding dual feature extraction methods.

Enlightened by the significant influence from feature extraction methods, we list all the extracted spectral ranges by the four methods for the ternary adulteration case in Figure 7. By a comparison between panels (a) and (b), it is found that CARS is able to grasp spectral ranges that comprise the characteristics for the compositional oil species: for instance, the 1265 cm^−1^ peak at which different oil species show relative differences in peak intensities. On the other hand, ICA fails to extract such characteristic features. CARS-ICA further narrows the selected spectral range and turns out to improve the final prediction performance. Such an improvement is possibly attributed to the further reduction in the redundancy in modeling inputs. ICA-CARS, on the other hand, ends up with the most inferior performance among all the methods. This is possibly due to the fact that the method has ruled out most of the characteristic spectral ranges by performing ICA at the first step, and the selected ranges are further narrowed by performing CARS afterwards, making ICA-CARS fail to provide an effective input for subsequent modeling. The above deduction is clearly supported by panel (d): for the isolated circles, which represent the output by ICA-CARS, none of them are characteristic for the compositional oil species. To provide a further insight into the role of feature extraction methods, Figure S6 presents the selected spectral ranges by CARS and ICA in the binary case. Unlike the ternary case, several selections of CARS and ICA overlap and both methods cover some spectral characteristics for the compositional oil species, which explains why both CARS and ICA are able to provide predictions with a similar level of accuracy.

For the models developed in this work, there is still a long way to go until we can realize their actual industrial application. However, within the scope of this work, we can still consider the time cost for different models and compare them in a more application-oriented perspective. The number of spectral variables serves as the indicator of the model’s complexity, and the total time duration to finish feature extraction and regression modeling serves as the indicator of the model’s time cost. The corresponding values are listed in Tables S6 and S7 for the binary and ternary case. Since very similar trends are demonstrated by both cases, here, we focus on the ternary case. Although feature extraction methods lower the complexity of the model, the time cost is different when the method varies. In our work, the CARS duration is roughly 40 s, in contrast to the ICA duration of nearly 100 s. Such a duration difference further leads to the contrast between CARS-ICA duration and ICA-CARS duration, that is, 54 s vs. 116 s. Based on previous contents, the CARS-ICA-PLSR model not only demonstrates the best performance, but also performs in a very time-saving manner. When switching from PLSR/BP to RF, the model duration is increased from tens of seconds to hundreds of seconds. Thus, the RF-based models not only demonstrate inferior performances, but also work in a more time-consuming way. For the BP- and PLSR-based models, it is the feature extraction duration that takes up the majority of the total duration. Thus, there is a general anti-correlation between the complexity and the total time cost. However, as indicated by our work, the lowering of complexity does not always lead to better performances (i.e., CARS-ICA-PLSR vs. ICA-CARS-PLSR); it is possible to achieve a quite satisfactory performance in a time-saving manner.

## 4. Conclusions

In this work, both binary and ternary camellia oil-targeted adulterations are analyzed quantitatively, using different feature extraction methods and regression algorithms. For camellia–rice blended-oil samples, both the ICA-BPNN and ICA-PLSR models have generally satisfactory prediction performances, demonstrating a high R^2^ and low RMSE. The performance for the RF-based models is somewhat inferior. The performance fluctuation when switching from ICA to CARS is not large. As for the camellia–corn–rice blended-oil samples, the best prediction is achieved by a PLSR model coupled with CARS-ICA dual feature extraction, demonstrating a high R^2^ for all the three compositional oil species. The performance difference among varying models is much larger in the ternary case than in the binary case. Such a difference is mostly rooted in the fact that CARS outstrips ICA in grasping the most characteristic spectral ranges in the ternary case, and the corresponding dual extraction method CARS-ICA further cuts off the redundancy and improves the subsequent regression modeling. The time costs of the models are also considered. It turns out that the CARS-ICA-PLSR model, which achieves the best performance in the ternary case, works in a very time-saving manner.

## Data Availability

The original contributions presented in this study are included in the article/Supplementary Materials. Further inquiries can be directed to the corresponding author.

## Supplementary Materials

The following supporting information can be downloaded at https://www.mdpi.com/article/10.3390/foods13244182/s1: Figure S1: (a–g) Raman spectra for camellia–rice blended-oil samples with varying adulteration concentrations. The adulteration concentrations are marked in the plots accordingly; Figure S2: Raman spectra for camellia–corn–rice blended-oil samples with varying total adulteration concentrations. The adulteration concentrations are marked in the plots accordingly; Figure S3: Prediction of adulteration concentration for camellia–rice blended-oil samples using CARS coupled regression models: regression results for (a) BPNN, (b) PLSR, and (c) RF; residual distribution for (d) BPNN, (e) PLSR, and (f) RF. In panels (d–f), the dash-dotted horizontal line are for eye guidance purposes; Figure S4: Prediction for camellia–corn–rice blended-oil samples using CARS-ICA-BPNN model: regression results for (a) camellia oil, (b) corn oil, and (c) rice bran oil; residual distributions for (d) camellia oil, (e) corn oil, and (f) rice bran oil. In panels (d–f), the dash-dotted horizontal lines are for eye guidance purposes; Figure S5: Prediction for camellia–corn–rice blended-oil samples using CARS-ICA-RF model: regression results for (a) camellia oil, (b) corn oil, and (c) rice bran oil; residual distributions for (d) camellia oil, (e) corn oil, and (f) rice bran oil. In panels (d–f), the dash-dotted horizontal lines are for eye guidance purposes; Figure S6: Feature extraction results for camellia- rice blended-oil samples using different methods: (a) ICA, (b) CARS; Table S1: Cumulative Variance by different number of principal components; Table S2: Evaluation metrics of regression models for camellia–rice blended-oil samples; Table S3: Evaluation metrics of regression models for predicting camellia oil concentration in camellia–corn–rice blended-oil samples; Table S4: Evaluation metrics of regression models for predicting corn oil concentration in camellia–corn–rice blended-oil samples; Table S5: Evaluation metrics of regression models for predicting rice bran oil concentration in camellia–corn–rice blended-oil samples; Table S6: Time cost (duration) and complexity (number of spectral variables) for predicting rice bran oil concentration in camellia–rice blended-oil samples; Table S7: Time cost (duration) and complexity (number of spectral variables) for predicting camellia oil concentration in camellia–corn–rice blended-oil samples; Table S8: List of abbreviations.
