# Supplementary material for: Quantitatively Detecting Camellia Oil Products Adulterated by Rice Bran Oil and Corn Oil Using Raman Spectroscopy: A Comparative Study Between Models Utilizing Machine Learning Algorithms and Chemometric Algorithms
Source: Foods. 2024 Dec 23;13(24):4182. doi: 10.3390/foods13244182 (PMC11675215; doi:10.3390/foods13244182)
Supplement: Supplementary file 1 [file foods-13-04182-s001.zip › foods-3345433-supplementary.pdf]

Figure S1. (**a-g**) Raman spectra for camellia-rice blended oil samples with varying adulteration concentrations. The adulteration concentrations are marked in the plots accordingly.

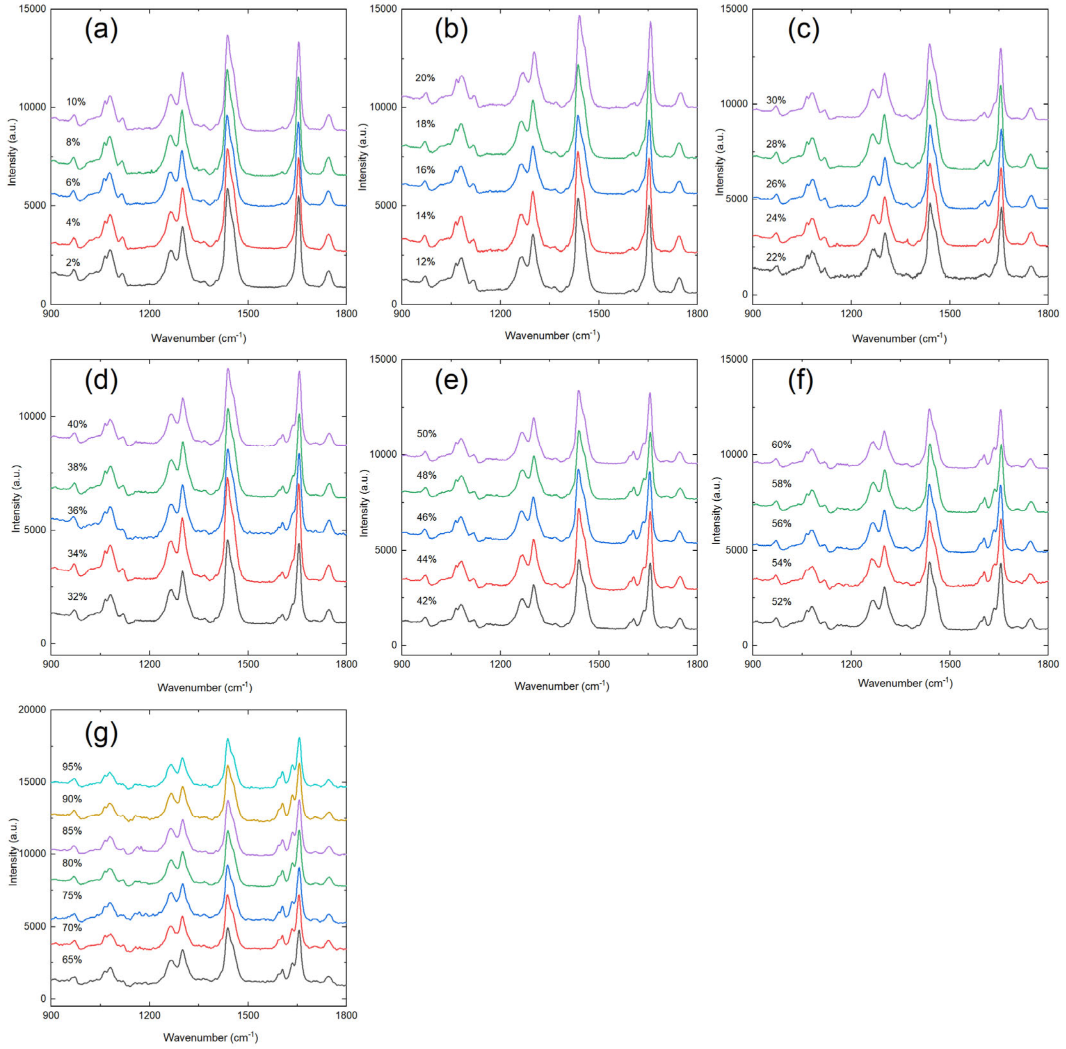

Figure S2. Raman spectra for camellia-corn-rice blended oil samples with varying total adulteration concentrations. The adulteration concentrations are marked in the plots accordingly.

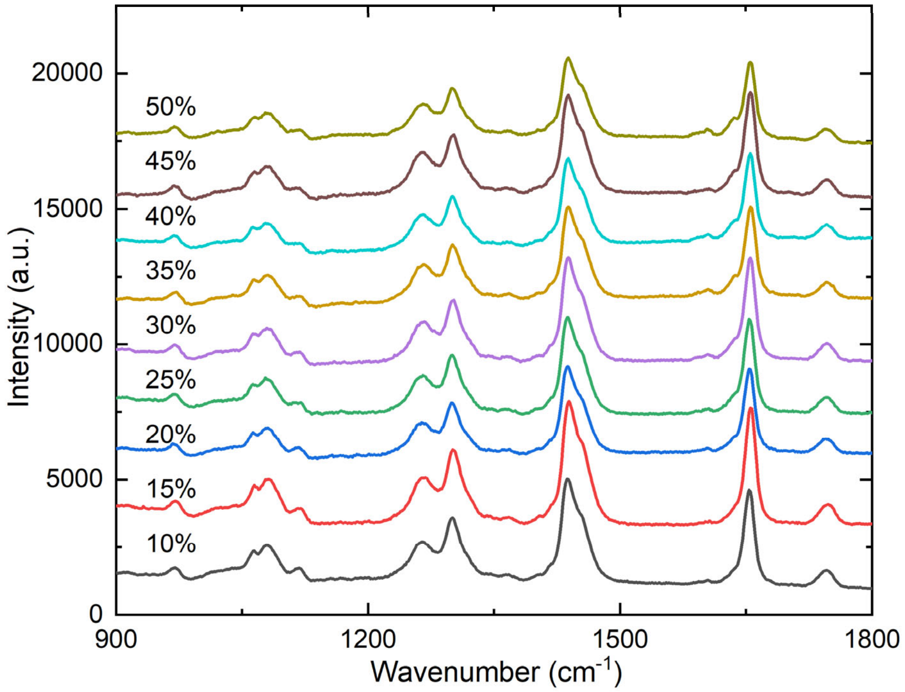

Figure S3. Prediction of adulteration concentration for camellia-rice blended oil samples using CARS coupled regression models: regression results for (**a**) BPNN, (**b**) PLSR, and (**c**) RF; residual distribution for (**d**) BPNN, (**e**) PLSR, and (**f**) RF. In panels (d-f), the dash-dotted horizontal line are for eye-guidance purposes.

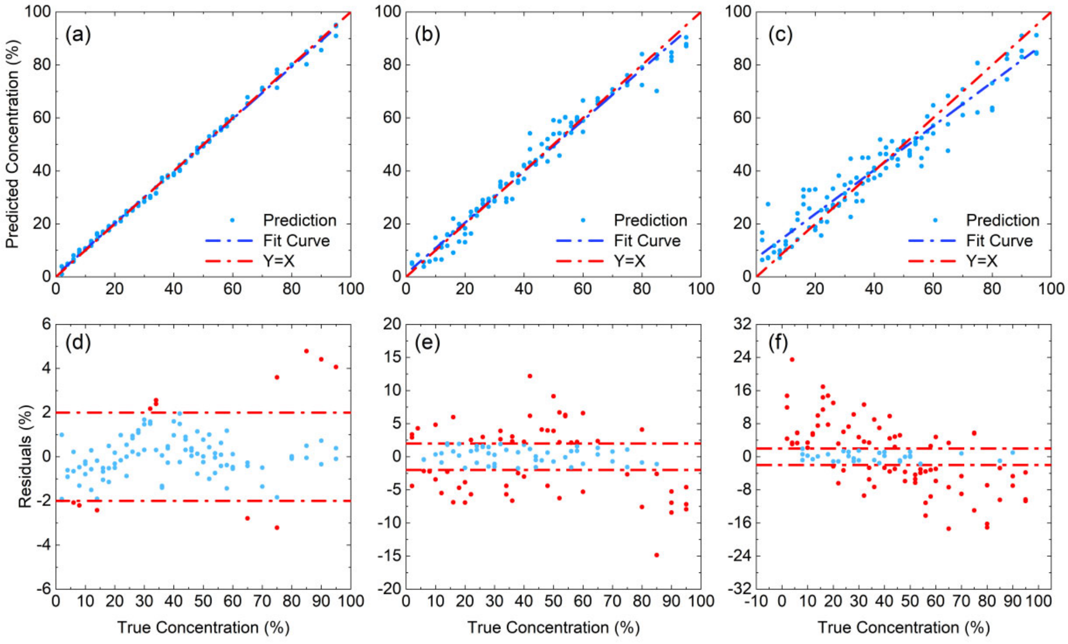

Figure S4. Prediction for camellia-corn-rice blended oil samples using CARS-ICA-BPNN model: regression results for (**a**) camellia oil, (**b**) corn oil, and (**c**) rice bran oil; residual distributions for (**d**) camellia oil, (**e**) corn oil, and (**f**) rice bran oil. In panels (d-f), the dash-dotted horizontal lines are for eye-guidance purposes.

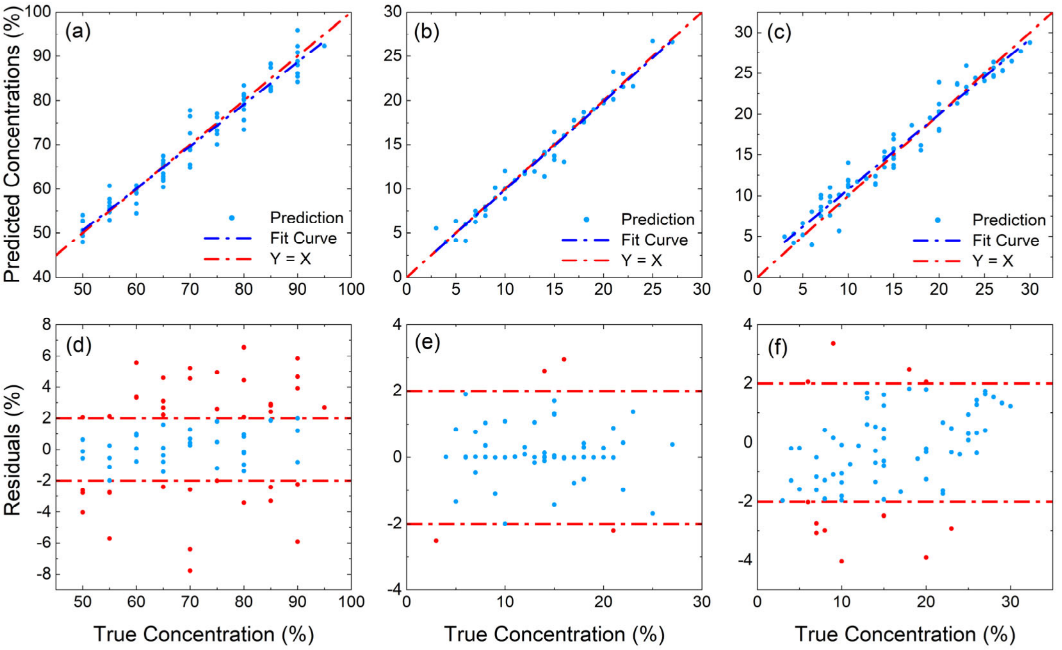

Figure S5. Prediction for camellia-corn-rice blended oil samples using CARS-ICA-RF model: regression results for (**a**) camellia oil, (**b**) corn oil, and (**c**) rice bran oil; residual distributions for (**d**) camellia oil, (**e**) corn oil, and (**f**) rice bran oil. In panels (d-f), the dash-dotted horizontal lines are for eye-guidance purposes.

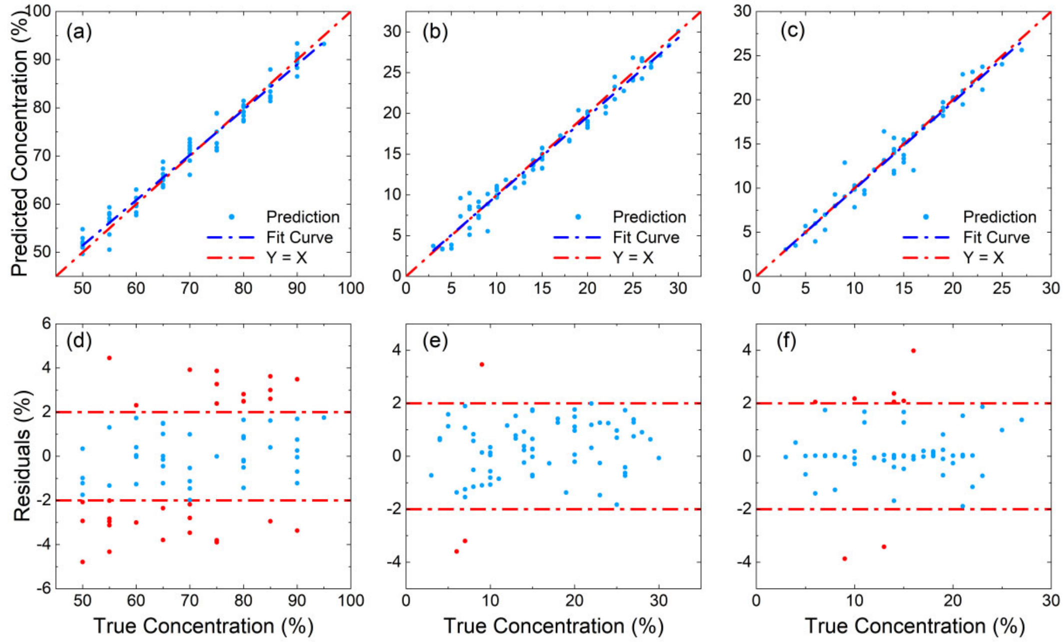

Figure S6. Feature extraction results for camellia- rice blended oil samples using different methods: (**a**) ICA, (**b**) CARS.

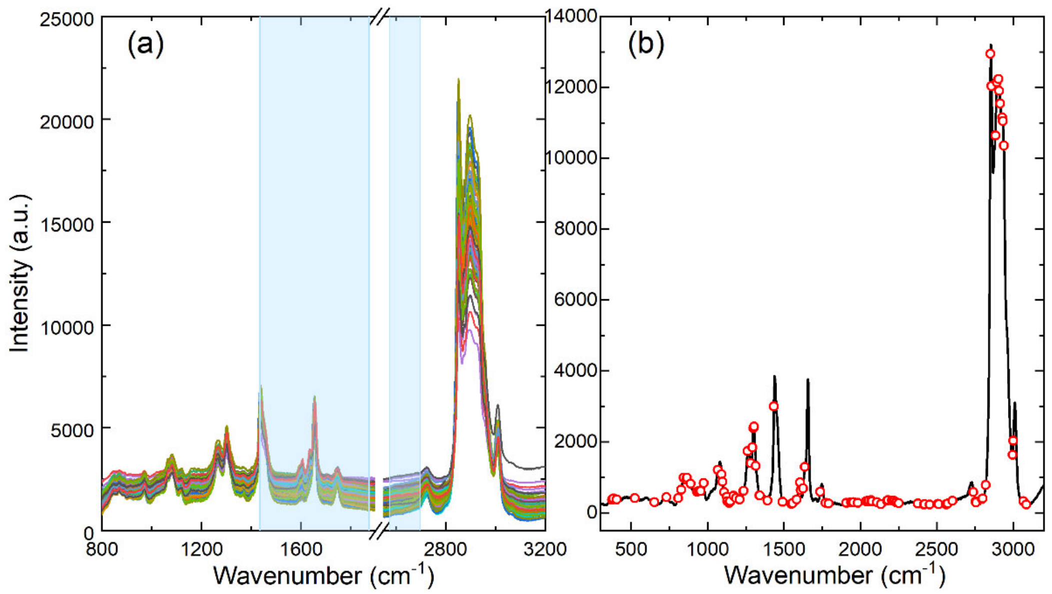

Table S1. Cumulative Variance by different number of principal components.

Principal Component Number	Eigenvalue	Percentage of Variance(%)	Cumulative(%)
1	1924.24643	99.44426	99.44426
2	7.29821	0.37717	99.82143
3	2.68536	0.13878	99.96021
4	0.31605	0.01633	99.97654
5	0.24336	0.01258	99.98912

Table S2. Evaluation metrics of regression models for camellia-rice blended oil samples.

Regression algorithm	Feature extraction method	Evaluation					
		R^2C	R^2P	R^2	RMSEC	RMSEP	RMSE
BPNN	ICA	0.9939	0.99368	0.99379	1.9358	2.1099	2.02285
PLSR		0.9975	0.94094	0.96922	1.2161	6.3923	3.8042
RF		0.9702	0.9244	0.9473	5.2633	5.8919	5.5776
BPNN	CARS	0.9951	0.975	0.98505	1.7151	4.1622	2.93865
PLSR		0.98734	0.94638	0.96686	2.8168	6.454	4.6354
RF		0.9313	0.9529	0.9421	7.2404	5.4685	6.35445

Table S3. Evaluation metrics of regression models for predicting camellia oil concentration in camellia-corn-rice blended oil samples.

Regression algorithm	Feature extraction method	Evaluation					
		R^2C	R^2P	R^2	RMSEC	RMSEP	RMSE
BPNN	ICA	0.6700	0.5245	0.5972	7.3177	9.129	8.2234
	CARS	0.9504	0.8877	0.9191	2.8888	4.3545	3.6217
	ICA-CARS	0.1864	0.1299	0.1582	11.4836	12.6277	12.0557
	CARS-ICA	0.9444	0.9447	0.9445	2.9238	3.1859	3.05485
PLSR	ICA	0.9321	0.6212	0.7766	3.4618	7.4757	5.4688
	CARS	0.9717	0.8699	0.9208	2.0964	4.8441	3.4703
	ICA-CARS	0.2354	0.1726	0.2040	10.8406	12.5429	11.6918
	CARS-ICA	0.9721	0.9634	0.9678	2.3187	2.3428	2.3307
RF	ICA	0.8604	0.8652	0.8628	6.2449	7.5394	6.8922
	CARS	0.944	0.886	0.915	4.5964	5.9175	5.2560
	ICA-CARS	0.8282	0.7781	0.80315	7.2328	7.0781	7.1555
	CARS-ICA	0.9717	0.96139	0.966525	2.1448	2.5342	2.3395

Table S4. Evaluation metrics of regression models for predicting corn oil concentration in camellia-corn-rice blended oil samples.

Regression algorithm	Feature extraction method	Evaluation					
		R^2C	R^2P	R^2	RMSEC	RMSEP	RMSE
BPNN	ICA	0.46037	0.34497	0.40267	7.0131	6.0245	6.5188
	CARS	0.7421	0.6343	0.6882	4.5936	5.4622	5.0279
	ICA-CARS	0.0338	0.0776	0.0557	9.2851	7.9023	8.5937
	CARS-ICA	0.98446	0.95455	0.969505	0.63449	1.3635	0.998995
PLSR	ICA	0.9963	0.5135	0.7549	0.17241	6.5117	3.342055
	CARS	0.86833	0.82815	0.84824	3.4063	3.5549	3.4806
	ICA-CARS	0.1511	0.045677	0.0984	8.1351	9.5439	8.8395
	CARS-ICA	0.99309	0.98476	0.988925	0.61429	0.90761	0.76095
RF	ICA	0.7798	0.753	0.7664	5.0542	5.7265	5.39035
	CARS	0.94396	0.88597	0.914965	4.5964	5.9175	5.25695
	ICA-CARS	0.7507	0.8172	0.78395	7.352	6.9679	7.15995
	CARS-ICA	0.98147	0.964853	0.9732	0.98853	1.6583	1.323415

Table S5. Evaluation metrics of regression models for predicting rice bran oil concentration in camellia-corn-rice blended oil samples.

Regression algorithm	Feature extraction method	Evaluation					
		R^2C	R^2P	R^2	RMSEC	RMSEP	RMSE
BPNN	ICA	0.34456	0.35333	0.348945	6.0367	4.0201	5.0284
	CARS	0.4561	0.4571	0.4566	5.2662	4.1439	4.70505
	ICA-CARS	0.3712	0.065	0.2181	5.3183	6.9147	6.1165
	CARS-ICA	0.96044	0.92145	0.940945	1.3665	2.3334	1.84995
PLSR	ICA	0.8821	0.40875	0.645425	2.388	4.6205	3.50425
	CARS	0.83773	0.45112	0.644425	2.5742	5.4684	4.0213
	ICA-CARS	0.29063	0.14988	0.220255	4.7907	8.0655	6.4281
	CARS-ICA	0.97058	0.98126	0.97592	0.77216	0.79957	0.785865
RF	ICA	0.8125	0.8403	0.8264	3.6186	2.7407	3.17965
	CARS	0.8671	0.826	0.84655	3.5392	2.2051	2.87215
	ICA-CARS	0.7404	0.7798	0.7601	4.3123	2.8997	3.606
	CARS-ICA	0.9615	0.9479	0.9547	1.0258	1.48	1.2529

Table S6. Time cost (duration) and complexity (number of spectral variables) for predicting rice bran oil concentration in camellia-rice blended oil samples. The algorithms are run by a regular laptop with the following configuration: Intel(R) Core(TM) i5-10210U, 8GB RAM and 256GB SSD.

Regression algorithm	Feature extraction method	Number of spectral variables	Feature extraction duration (s)	Modeling duration (s)	Total duration (s)
BPNN	/	1934		141.627475	141.627475
	ICA	380	80.210116	13.255057	93.465173
	CARS	102	60.309124	5.922844	66.231968
PLSR	/	1934		4.67658	4.67658
	ICA	380	80.210116	2.667561	82.877677
	CARS	102	60.309124	1.507114	61.816238
RF	/	1934		598.245278	598.245278
	ICA	380	80.210116	441.150655	521.360771
	CARS	102	60.309124	381.049474	441.358598

Table S7. Time cost (duration) and complexity (number of spectral variables) for predicting camellia oil concentration in camellia-corn-rice blended oil samples. The algorithms are run by a regular laptop with the following configuration: Intel(R) Core(TM) i5-10210U, 8GB RAM and 256GB SSD.

Regression algorithm	Feature extraction method	Number of spectral variables	Feature extraction duration (s)	Modeling duration (s)	Total duration (s)
BPNN	/	1934		98.13487	98.13487
	ICA	284	99.585532	15.606808	115.19234
	CARS	44	40.331264	8.50749	48.838754
	ICA+CARS	11	115.977688	10.848021	126.825709
	CARS+ICA	18	53.861053	16.934077	70.79513
PLSR	/	1934		8.156823	8.156823
	ICA	284	99.585532	2.764287	102.349819
	CARS	44	40.331264	3.102079	43.433343
	ICA+CARS	11	115.977688	2.51694	118.494628
	CARS+ICA	18	53.861053	3.327756	57.188809
RF	/	1934		398.56732	398.56732
	ICA	284	99.585532	167.979836	267.565368
	CARS	44	40.331264	265.034759	305.366023
	ICA+CARS	11	115.977688	174.9192	290.896888
	CARS+ICA	18	53.861053	139.677742	193.538795

Table S8. List of abbreviations.

Savitzky-Golay	S-G
Multiplicative Scatter Correction	MSC
Independent Component Analysis	ICA
Competitive Adaptive Reweighing Sampling	CARS
Principal Component Analysis	PCA
Levenberg Marquardt	LM
Partial Least Squares Regression	PLSR
Backpropagation Neural Network	BPNN
Random Forest	RF
Linear Discriminant Analysis	LDA
Coefficient of Determination	R^2
Root Mean Square Error	RMSE
